# Dermatological Opportunistic Infections in HIV Seropositive Patients: An Observational Study

**DOI:** 10.7759/cureus.16852

**Published:** 2021-08-03

**Authors:** Sanket D Basida, Brinda Basida, Nirav Zalavadiya, Arti P Trivedi

**Affiliations:** 1 Department of Internal Medicine, Pandit Deendayal Upadhyay Medical College, Rajkot, IND; 2 Department of Internal Medicine, Detroit Medical Center Sinai-Grace Hospital, Detroit, USA

**Keywords:** opportunistic infections, dermatologic manifestations, muco-cutaneous diseases, cd4+ cell count, hiv dermatoses

## Abstract

Background and objective

In developing countries, the dermatological manifestation of the human immunodeficiency virus (HIV) has a high prevalence. Apart from the systemic infection that ensues HIV, skin manifestations form a major part of the disease burden. They can present with atypical forms, and necessary tools for diagnosis may not be available in rural and remote parts of the country. Hence, they can stay misdiagnosed or undiagnosed, contributing to the morbidity of the patients. We attempted to enumerate the dermatologic opportunistic infections (OIs) in Rajkot city, Gujarat, India, in order to disseminate knowledge regarding the same.

Material and methods

It is a retrospective observational study. A total of 253 patients under treatment for HIV/acquired immunodeficiency syndrome (AIDS) at the ART Center (anti-retroviral therapy center) from 2011 to 2019 were included. The data recorded in the registry during the above-mentioned period were utilized in the study. The diagnoses of OIs were made clinically by multiple health care providers experienced in the field.

Result

Two hundred twenty-seven (227) of 253 (89.72%) of the patients had some form of dermatologic OI during the course of their treatment. Overall, fungal infections (33.03%) were most common, followed by bacterial infections (28.18%) and viral (14.55%) infections. Among the non-infectious causes, cheilitis/angular stomatitis topped the list. Among the STDs, herpes was the most common skin manifestation seen with a 10.57% prevalence. The CD4+ cell count for fungal infection ranged from 353-467 and was seen in stage 2 of the disease course. Bacterial infections were seen mainly during the early and middle stages of the disease while viral infections were most prevalent in stage 2 of the disease.

Conclusion

Skin manifestations can be useful clinical predictors of the disease stage, especially in resource-limited settings and in developing countries. They can present with unusual and atypical forms. Hence, knowledge about the prevalence of these OIs in a particular geographical area can be very useful for physicians in treating them and decreasing the disease burden.

## Introduction

The burden of skin diseases in human immunodeficiency virus (HIV) patients in developing countries is huge. It was reported that approximately 90% of people living with HIV have skin changes and symptoms during the course of their disease [1]. Skin findings are regarded by WHO as useful in assessing the severity of HIV infection in patients in a resource-limited environment [2].

Knowledge of the skin and mucosal signs of HIV/acquired immunodeficiency syndrome (AIDS) is important. Opportunistic infections (OIs) are usually the first manifestation of HIV, ensuring early diagnosis and prompt treatment, and reveals complications, as HIV causes atypical and severe presentations of these conditions [3]. Therefore, those involved in the health care of HIV patients must know the type, pattern, and prevalence of skin diseases in their locality [4-5].

These include Herpes zoster, varicella-zoster, bacillary angiomatosis, candidiasis, Kaposi sarcoma, common warts, and many others. They are related to low CD4+ cell counts and immunosuppression. But drug eruptions or inflammatory skin disease are due to health alteration of the immune system in the skin of HIV-positive patients [6].

HIV attacks the helper/inducer T cells (CD4+ cells), resulting in syncytial formation and lysis with slow but progressive destruction of this cell population. Therefore, it is a reliable prognostic indicator of immune response to therapy [7-8]. In general, the CD4+ cells (%CD4+ or absolute count) progressively decreases as HIV disease advances [9]. As very few studies have been done in our locality, we have attempted to do the same to increase awareness among physicians regarding the prevalence of these dermatologic OIs.

## Materials and methods

The study design is a retrospective observational study. The data of patients from January 2011 to April 2019 enrolled at the ART Centre, Rajkot, Gujarat, India, were collected. The study population consists of 253 HIV seropositive patients with AIDS-defining illnesses, out of which 227 patients had mucocutaneous OIs. The diagnosis of the dermatological manifestation was made clinically. We included all seropositive patients with OIs from all age groups. The only exclusion criteria in our study were patients with missing or incomplete data. The data were collected, sorted, and analyzed by the authors in this study. It is a descriptive study, and no statistical analysis was performed. IRB approval from the ethics committee and informed consent were obtained from all the patients in the study.

## Results

**Figure 1:**
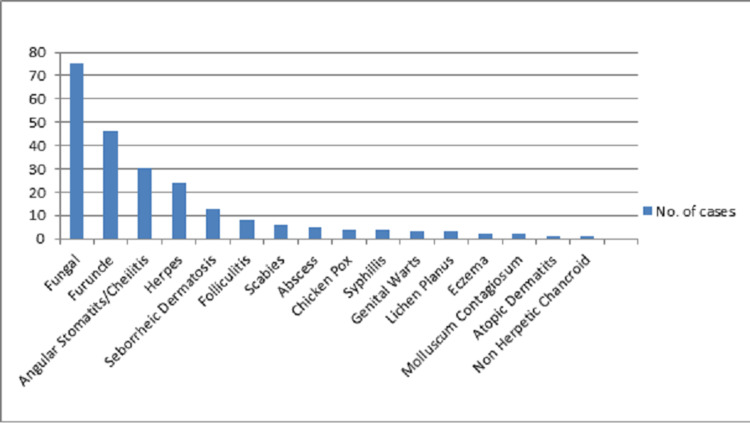
Graphical presentation of the prevalence of dermatological opportunistic infections

Overall fungal infections (dermatophytosis, candida) accounted for most of the opportunistic infections (33.03%). This is because cell-mediated immunity is the major mechanism of controlling normal fungal commensals from flourishing. In HIV patients, cell-mediated immunity is down, which allows these fungal commensals to grow and cause infection. Among others, furuncle (20.26%) and angular stomatitis/cheilitis (13.21%) accounted for most dermatologic manifestations. The impaired skin barrier and severe neutropenia may be the causes of such a finding. A summary of the prevalence of all the dermatologic OIs can be found in Table 1 and the graphical presentation in Figure 1.

**Table 1:** Prevalence of dermatological opportunistic infections in the study population *n is the total number of patients with dermatological manifestations.

Infectious Disorder	Total(n=227)*
Fungal	75(33.03%)
Furuncle	46(20.26%)
Angular Stomatitis/Cheilitis	30(13.21%)
Herpes	24(10.57%)
Seborrheic Dermatitis	13(5.72%)
Folliculitis	8(3.52%)
Scabies	6(2.64%)
Abscess	5(2.2%)
Chicken Pox	4(1.76%)
Syphilis	4(1.76%)
Genital Warts	3(1.32%)
Lichen Planus	3(1.32%)
Eczema	2(0.9%)
Molluscum Contagiosum	2(0.9%)
Atopic Dermatitis	1(0.44%)
Non Herpetic Chancroid	1(0.44%)

The number of dermatologic manifestations progressively increased as the CD4+ cell count went down. Chickenpox and furuncle were found in stage 1 of the disease with a mean CD4+ cell count of >500 cells/mm^3^. In stages 2 and 3 of the disease, the majority of the dermatoses were found, including infectious and non-infectious causes. A summary of the same findings can be seen in Table 2 and Table 3.

**Table 2:** Co-relation of dermatological opportunistic infections with CD4+ cell count

Infectious Disorders	CD4+ Cell Count Range (cells/mm^3^)	Mean CD4+ Cell Count (cells/mm^3^)
Chicken Pox	558-874	600
Scabies	233-275	250
Furuncle	510-660	550
Folliculitis	200-250	220
Seborrheic Dermatitis	360-480	400
Fungal	353-467	400
Molluscum Contagiosum	100-200	150
Atopic Dermatitis	290	290
Herpes	371-410	390
Angular Stomatitis/Cheilitis	288-320	300
Genital Warts	270-300	280
Eczema	367-455	400
Abscess	100-550	250
Lichen Planus	250-323	270
Syphilis	200-330	300
Non-Herpetic Chancroid	450	450

**Table 3:** Immunologic staging of dermatological opportunistic infections

Dermatological Manifestations	CD4+ Cell Count (cells/mm^3^)	Immunologic Staging(WHO)
Chickenpox	More than or equal to 500	Stage 1
Furuncle		
Seborrheic Dermatitis	350 to 499	Stage 2
Fungal Infection		
Herpes		
Eczema		
Non-Herpetic Chancroid		
Angular Stomatitis/Cheilitis	200 to 349	Stage 3
Atopic Dermatitis		
Genital Warts		
Scabies		
Folliculitis		
Lichen Planus		
Syphilis		
Molluscum Contagiosum	Less than 200	Stage 4

**Figure 2:**
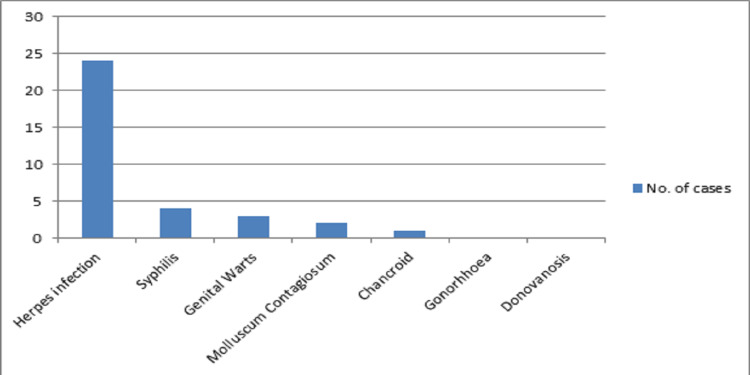
Graphical presentation of the prevalence of sexually transmitted diseases

Among all the sexually transmitted diseases (STDs), herpes accounts for most of the dermatoses followed by a few cases of syphilis, genital warts, and molluscum contagiosum. We also found a single case of chancroid in our study. A summary of these findings is represented in Table 4 and graphically presented in Figure 2.

**Table 4:** Prevalence of sexually transmitted diseases as part of opportunistic infections

Sexually Transmitted Diseases	Total (n=227)
Herpes Infection	24 (10.57%)
Syphilis	4 (1.76%)
Genital Warts	3 (1.32%)
Molluscum Contagiosum	2 (0.88%)
Chancroid	1 (0.44%)
Gonorhhoea	0 (0%)
Donovanosis	0 (0%)

## Discussion

The introduction of highly active antiretroviral therapy (HAART) has drastically changed the natural course of HIV and decreased the incidence of OIs by decreasing the viral load and increasing the CD4+ cell count [10-11]. The immune restoration syndrome that arises due to recovery of the immune system by HAART has been implicated in the development of previously dormant infections, e.g. herpes zoster, mycobacterial infections, etc. [12-13]. These drugs are also responsible for inflammatory adverse drug reactions [14]. In this study, we focused on mucocutaneous OIs regardless of their HAART status. The tertiary care center where this study was conducted was the referral site for all the surrounding rural areas and, therefore, the study population was a good representation of the actual population.

Out of 253 seropositive patients, 227 (89.72%) patients had some form of dermatologic OIs at some point during their treatment. It was similar to studies done by Singh et al. (87.6%) [15], Jeffrey et al. (86%) [16], and Pitche et al. (82.5%) [17], whereas a study done by Spira et al. [18] showed that it is relatively lower (65.3%).

We found that the most common dermatoses in HIV were due to infection (77.36%), which is slightly higher than studies done by Oninla (65.2%) [19] and Salami et al. (64.2%) [20], who also found infection as the most common cause. Infections start to develop in the early stages of HIV while progressing to more diffuse forms in the later stages of the disease [21-22], which was seen in our study.

Overall fungal infection (candida, dermatophytosis) (33.03%) was the most common mucocutaneous manifestation. Similar findings were also seen in studies done by Singh et al. [15] and Spira et al. [18]. Among other infectious manifestations, bacterial infections (28.18%) and viral infections (14.55%) were the most common. These findings were comparable to studies done by Oninla (50%, 12%, and 3.2%) [19] for the prevalence of fungal, viral, and bacterial dermatologic manifestations, respectively, and the Salami (37% and 24.3%) [20] prevalence for fungal and viral manifestations.

Seborrheic dermatitis (5.72%) is frequently found to be the most common dermatologic manifestation but that was not seen in our study [4,15]. In the present study, angular cheilitis/stomatitis was found to be the most common non-infectious dermatologic manifestation at 13.21%.

Among all the sexually transmitted diseases, herpes infection accounted for most of them (6%), which was comparable to other studies [19]. Other STDs found in the present study were syphilis (1.76%), genital warts (1.32%), molluscum contagiosum (0.88%), and chancroid (0.44%).

The co-relation to CD4+ cell count that we observed in our study co-related well with the World Health Organization (WHO) staging of the disease. As the disease progressed, the number of dermatoses observed increased. Similar results were seen in studies done by Wiwanitkit [23] and Nnoruka et al. [22]. Sivayathorn et al. mentioned that the number of dermatoses found in stages 2 and 3 was more as compared to stage 1 [24].

Fungal infections were predominantly seen in stage 2 of the disease in the present study, but they were not exclusive to these stages. Sharma et al. [25] and Goh et al. [26] reported <200 cells/mm^3^ CD4+ cell count for oral candidiasis. Oral candidiasis particularly extending to the esophagus is associated with severe immunosuppression and therefore these are good clinical indicators of advanced HIV infection [19].

Bacterial infections were found to be prevalent in the early and middle part of the clinical staging of HIV with CD4+ cell count ranging from 200-660 cells/mm^3^. Nnoruka et al. found bacterial infections in the CD4+ cell count range of 200 to 500 cells/mm3.

Viral infections were found in all the stages of the disease with herpes being most prevalent in the second stage. WHO has also classified it as a second-stage disease. In the present study, genital warts were found in those with stage 3 of HIV. WHO, however, has classified it in stage 2. Mawenzi et al. have mentioned in their study that genital warts are more prevalent in patients with a CD4+ cell count of >300 cells/mm^3^ [27].

Seborrheic dermatosis was found to be in stage 2 in the present study, which is similar to where WHO has listed the disease. This finding is similar to the ones found by Oninla [19] and Nnoruka [22].

## Conclusions

Mucocutaneous manifestations of HIV are not only the cause of morbidity and serious concern to the patients but are of great help in the early identification of cases of HIV patients. Many such manifestations are a marker of AIDS. Both infectious and noninfectious causes are responsible for morbidity in these patients. These manifestations can be taken care of with appropriate precautions and medications. There is a definite correlation between CD4+ cell count and mucocutaneous manifestations of HIV. In the search for reliable clinical indicators for the management of HIV/AIDS in resource-poor settings, mucocutaneous disorders of HIV should be considered among key clinical indicators for the prediction of underlying immune status, disease progression, and possible complications of highly active antiretroviral therapy.
